# Impaired trial-by-trial adjustment of cognitive control in obsessive compulsive disorder improves after deep repetitive transcranial magnetic stimulation

**DOI:** 10.1186/s12868-015-0205-z

**Published:** 2015-10-09

**Authors:** Mandana Modirrousta, Benjamin P. Meek, Jitender Sareen, Murray W. Enns

**Affiliations:** Department of Psychiatry, University of Manitoba, Winnipeg, MB Canada; Saint Boniface General Hospital, M4-McEwen Building, 409 Taché Avenue, Winnipeg, MB R2H 2A6 Canada

**Keywords:** Obsessive compulsive disorder, Error monitoring, Transcranial magnetic stimulation

## Abstract

**Background:**

Adaptive decision making requires the adjustment of behaviour following an error. Some theories suggest that repetitive thoughts and behaviours in obsessive compulsive disorder (OCD) are driven by malfunctioning error monitoring. This malfunction may relate to demonstrated hyperactivity in the medial prefrontal cortex (mPFC), including the dorsal anterior cingulate cortex. In this study, we measured aspects of error monitoring in individuals with OCD and administered deep low frequency repetitive transcranial magnetic stimulation (rTMS) in an attempt to modulate error monitoring capacity.

**Methods:**

For this pilot study, ten OCD patients and 10 aged-matched healthy controls completed modified versions of the Eriksen Flanker task before and after one session of deep 1 Hz rTMS (1200 pulses) over the mPFC (Brodmann areas 24 and 32). OCD patients received nine additional sessions of daily rTMS to assess their clinical response. Flanker tasks were repeated with patients post-treatment.

**Results:**

Overall error rates were higher for patients compared to controls. When subjects were asked to report their errors, OCD patients were able to report fewer of their errors than the control group. In contrast to controls, patients did not demonstrate a normal post-error slowing (PES) phenomenon. This abnormal PES was mainly driven by abnormally slow response times (RTs) following correct responses rather than a failure to slow down after errors. Patients’ symptoms and slowed RTs following correct responses improved after ten sessions of rTMS.

**Conclusions:**

Certain aspects of error monitoring, namely conscious error report and post error slowing, are impaired in OCD. These impairments can at least be partly corrected by 1 Hz deep rTMS over the mPFC. Simultaneous improvement of OCD symptoms by this method might suggest a correlation between error monitoring impairment and OCD pathophysiology.

Trial registration: ClinicalTrials.gov NCT02541812; 09/02/2015

## Background

Phenomenologically, patients with Obsessive Compulsive Disorder (OCD) often exhibit an intense fear that incorrect acts may have serious or harmful consequences, leading to ritualistic, undesirable and anxiety provoking behaviours [1]. The persistence of this fear despite a lack of objective evidence for the presence of adverse consequences led to the proposal of an ‘action monitoring’ deficit in the psychopathology of these patients [1, 2]. The theory of abnormal action monitoring in OCD was further supported by subsequent works [2–4], which found a higher error-related negativity (ERN) in response to errors in OCD patients, regardless of the OCD subtype, compared to controls. ERN is a component of the event-related potential (ERP) and is characterized by a sharp negative signal which begins 50–100 ms following an error [2]. The enhanced ERN amplitude is independent of symptom states and consistently reported in adults and children with OCD, as well as in the unaffected first-degree relatives of individuals with OCD [4–6]. Source localization studies have identified the medial prefrontal cortex (mPFC) especially the dorsal anterior cingulate cortex (dACC) as a candidate generator of the observed ERN [7, 8].

Neuroimaging studies have revealed that neural activations in a network that responds to error commission in healthy people functions abnormally in OCD patients [9–11]. In healthy adults, errors activate a specific neural network that includes posterior medial frontal cortex (pMFC)/dorsal anterior cingulate cortex (dACC) and bilateral anterior insula/frontal operculum including regions of posterolateral orbitofrontal cortex (OFC) [2, 4, 12]. Neural activity in this circuit appears to be abnormal in OCD patients at rest [13], during symptom provocation [14], and when performing various cognitive tasks including error detection [15].

Despite these findings, the functional significance of heightened dACC and enhanced ERN in OCD are still debated. Some theories suggest that ERN reflects an error detection signal, and OCD patients with higher ERN perhaps exhibit an over-active error signal in response to mistakes [2]. Others speculate that ERN can be interpreted as a signal that triggers behavioural adjustment to improve performance and prevent future errors [16]. The correlates of these imaging and electrophysiological findings to patients’ actual performances in error monitoring tasks are inconsistent. OCD patients’ accuracy and reaction times were reported to be largely normal in the standard cognitive tasks that elicited abnormal ERN [12]. One study in pediatric OCD showed that patients failed to exhibit post-error slowing (PES) and post-conflict adaptation [17] while another study in adult OCD showed prolonged PES [12]. In the present study, we sought to clarify the inconsistencies in these reports by using a behavioural task that could parse out different components of error processing including error production, post-error slowing, error reporting, and error correction. We hoped to better elucidate which aspects of the error monitoring process might be impaired in OCD.

Furthermore, we wanted to know whether modulation of activity in the mPFC by low frequency repetitive transcranial magnetic stimulation (rTMS) can improve patients’ error monitoring ability. In recent decades, rTMS has increasingly been used to identify the function of localized brain regions. rTMS is a unique approach that allows relatively easy and painless central nervous system (CNS) stimulation. When the induced magnetic field is above a certain threshold, and is directed in an appropriate orientation relative to the brain’s neuronal pathways, localized axonal hyperpolarization or depolarization occurs. This in turn inhibits or activates the neurons in the relevant brain structure, respectively. In general, it is widely believed that high frequency (≥5 Hz) rTMS increases cortical excitability whereas low frequency (1 Hz) stimulation results in decreased cortical excitability [18, 19]. By inhibiting neuronal activity, low frequency rTMS induces temporary partial functional lesions in the brain and disrupts neural activity within a relatively localized cortical area [20–22]. A double-cone coil with large angled wings has been developed to modulate deeper cortical regions such as the dACC [23, 24]. A recent positron emission tomography (PET) study revealed that frontal TMS using a double-cone coil can indeed alter activity in these deeper structures [25]. A study using healthy subjects showed that creating a functional temporary lesion over the medial prefrontal cortex including the dACC using a double cone coil led to a decrease in the numbers of corrected errors in the Eriksen Flanker Task [26]. Other studies have demonstrated that rTMS over the midline frontal cortex selectively impairs perception of angry faces and Stroop task performance in volunteers [27, 28], illustrating the ability of deep coils to stimulate ACC.

In the present study, we used modified versions of Eriksen Flanker tasks that tested different aspects of error monitoring, i.e. post-error slowing, error report and error correction [29], to determine which, if any, components of error processing are affected by OCD. Given the involvement of mPFC and dACC in error monitoring and the fact that this same network shows altered functioning in individuals with OCD, we then sought to investigate whether modulation of this region could improve error monitoring performances in an OCD group. Specifically, we hypothesized that inhibition of the dACC using low frequency rTMS with a double-cone coil could improve performance by helping to dampen an overactive neural response to error production. Finally, we studied whether patients’ symptoms were correlated with their performance in error monitoring tasks before or after rTMS.

## Methods

Ten adult OCD patients and ten demographically matched healthy control subjects (CTL) were recruited for this pilot study. Patients were recruited from out-patient anxiety clinics at Health Sciences Centre in Winnipeg or through advertisements posted on local bulletin boards and OCD Manitoba Brochures. Eligible patients had to be taking either no medication or only one selective serotonin reuptake inhibitor (SSRI) or serotonin noradrenaline reuptake inhibitor (SNRI) to minimize potential variation in drug effect. In addition, as part of the study, participants were instructed not to make any adjustment to their medications during the study period (i.e. at least 2 weeks prior to rTMS and 4 weeks after the last session of rTMS). Eighteen adult OCD patients were screened for eligibility. Of those, five patients had a comorbid psychiatric disorder and three had medications other than SNRI or SSRI and were not enrolled. The remaining ten adult OCD patients met our eligibility criteria and were enrolled in the study. Of the enrolled patients, three were taking fluoxetine, two were taking sertraline, two were taking venlafaxine, and three were on no medication.

Structured psychiatric interviews using the Mini-International Neuropsychiatric Interview (M.I.N.I.) were performed by either a trained research assistant or the principal investigator (MM). Exclusion criteria included: any axis I psychiatric disorders other than OCD (e.g. including active alcohol or substance abuse, Mood Disorders, Psychotic Disorders, other Anxiety Disorders, etc.); neurological diseases or head injury; pregnancy; and implanted metal clips. Inclusion criteria included: patients with symptomatic OCD aged 18–65 years-old. The Yale-Brown Obsessive Compulsive Scale (Y-BOCS) [30] was administered by the MD clinician to assess baseline OCD symptoms. The clinician who administered the Y-BOCS was different from each patient’s treating physician and blind to the type of treatment OCD patients received. Although no cut-off score was used for inclusion/exclusion criteria, all eligible recruited participants except for one had a baseline Y-BOCS score above 20 (minimum Y-BOCS = 17, maximum Y-BOCS = 27). In addition, all subjects had failed to respond to at least one treatment intervention before participating in this study. All participants completed the Beck Depression Inventory—a self-reported questionnaire profiling depressive symptoms [31], the *Beck Anxiety Inventory*—a self-reported questionnaire profiling anxiety symptoms [32], and a version of the National Adult Reading Test (NART)—a quick test of estimating verbal intelligence. The study was approved by the University of Manitoba Health Research Ethics Board. All participants gave written informed consent in accordance with the principles set out in the Declaration of Helsinki and the stipulations of the local Institutional Review Boards. Participants received a nominal fee as compensation for their time and effort.

### Tasks

Participants completed three versions of the Eriksen Flanker task, each consisting of 400 trials, to measure post-error slowing, error correction, and error reporting, respectively. All participants performed the tasks in the same order. In all versions, subjects viewed a series of five arrows on a laptop computer screen and were instructed to indicate the direction of the central arrow by pressing the left or right response key with the index and middle fingers of their preferred hand as quickly and accurately as possible. An equal number of congruent (<<< or >>>) and incongruent (<> < or > <>) stimuli were presented, randomly intermixed, in each block of 400 trials. The first, “standard”, version required only a single response. The measure of interest was post-error slowing, i.e. the mean reaction time (RT) of correct trials that followed error trials compared with correct trials that followed correct trials. The second, “error reporting”, version required participants to push a separate key (with their other hand) on trials in which they believed they had made an error. The third, “error-correction”, version had subjects make a second, corrective response by pushing the alternate response key as quickly as possible if they believed their first response was an error.

Participants viewed each stimulus for 100 ms and subsequently had a maximum of 1000 ms in which to respond. The response period was terminated as soon as a response was registered. A secondary response period of a maximum of 500 ms for the “error correction” blocks and a maximum of 1000 ms for the “error reporting” blocks was added following initial response in order to allow adequate time to record the second response, if necessary. These secondary response periods were terminated as soon as a response was registered. In all conditions, a blank screen was displayed for 200 ms following the final response (or at the end of the pre-defined response period in the case of no response) before presentation of the next stimulus. A feedback screen was presented after every 20 trials. The feedback screen informed the participant of their average response time over the previous 20 trials, and text reminded them to continue to respond quickly and accurately. The purpose of this feedback was to encourage participants to perform the task as quickly as they could.

All OCD patients performed the tasks three times: during the intake session (within 1 week prior to rTMS), immediately after one session of rTMS (within 15 min following rTMS) and after ten sessions of rTMS (within 15 min following the tenth rTMS session). Given that the offline effect of one session of rTMS is thought to be short-lived (lasting minutes to hours), we had subjects perform the tasks as soon as possible following stimulation. A maximum of 15 min was required to set up participants with the computerized tasks following rTMS.

All CTL subjects performed the task during the intake session and immediately after one session of rTMS (within 15 min following rTMS). CTL subjects received only one session of rTMS. The OCD patients received ten sessions of rTMS in total to assess their clinical improvement in addition to their task performances after rTMS treatment.

### TMS

The rTMS paradigm consisted of eight short trains of 1 Hz magnetic pulses, each consisting of 150 pulses (overall 1200 pulses in one session) using the double-cone coil at 110 % intensity of the individual resting motor threshold. Patients received a total of ten sessions of rTMS. Sessions took place on consecutive business days (Monday through Friday), such that each patient’s treatment schedule covered a total of 12 days. The double-cone coil uses two angled windings to improve coupling to the head, increasing its effective stimulating power to relatively deep brain areas. This coil does not have a built-in a cooling system, so 2-min intervals between all stimulation trains, plus a 10-min interval after the fourth train were required to avoid coil over-heating. To measure the resting motor threshold in each subject single pulse TMS was delivered. Motor thresholds were established using the criterion of lowest intensity of stimulation that resulted in an electric pulse [a spike above 50 µV observed using electromyography (EMG)]. EMG was connected to participants’ toes to record the activity of their extensor hallucis brevis muscle following stimulation of the midline toe and leg area of primary motor cortex [33, 34]. The motor homunculus places toe representation in the depth of the central sulcus at a level anatomically close to the cingulate cortex. The location that produced the largest EMG pulse was identified and then stimulation intensity was reduced until 3/5 pulses produced a signal wave in the recording EMG.

The coordinates of the primary motor cortex for toe as well as the target area (anterior cingulate cortex, BA 24 and 32) were co-registered with the individual’s localizer images from a high-resolution 3-dimensional (3D) T1-weighted MRI scan using the BrainSight™ 2 navigator (Roch M. Comeau; http://www.rogue-resolutions.com/neuronavigation/brainsight, Montreal, QC). These images were normalized using MNI ATLAS coordinates and were superimposed with an overlay containing Brodmann areas (BAs). Using these programs, the TMS stimulation positions were accurately identified.

We used a Magstim Rapid 2 TMS system with its double-cone coil from Magstim Co. (http://www.magstim.com, UK) to stimulate the target area (BAs 24, 32). Hayward et al. showed that regional blood flow was significantly changed in the ACC (equivalent to BA 24) after double cone coil rTMS over the medial prefrontal cortex, indicating the efficacy of the double-cone coil for stimulation of deep cortical regions [25]. The ‘double-cone’ coil used in this study consists of two rings, each with a diameter of 110 mm, positioned at an angle of 95° to one another. This configuration allows for better coupling of the coil to the skull and the stimulation of deeper brain areas compared to a conventional ‘figure-eight’ coil. Brainsight software allows for the precise registration of a coil’s hotspot through its calibration procedure, in which a designated marker on the proprietary calibration block is aligned with the demarcated hotspot on the coil. Following calibration, Brainsight displays the coil as an arrow, which indicates both the location of the coil hotspot (tip of the arrow) and the direction of magnetic field penetration into the cortex (angle of the arrow shaft).

## Results

Demographic information and baseline symptom measurements are summarized in Table 1. There was no significant difference between the groups with regard to age [*t*(18) = 0.15; *p* > 0.05], education [*t*(18) = 1.5, *p* > 0.05] or NART IQ estimate [*t*(18) = 1.38; *p* > 0.05]. Patients’ scores on the BDI and BAI scales were significantly higher than those of CTLs [BDI: *t*(18) = −1.7, *p* < 0.05, BAI: *t*(18) = −2.5, *p* < 0.05]. However, based on structured neuropsychiatric interviews, none of the patients met the criteria for a mood disorder. The increase in self-reported anxiety symptoms in the patient group is in line with high anxiety levels in OCD populations. The YBOCS scores indicate that the patients’ symptom severity was moderate on average.

**Table 1:** Demographic information [mean (SD)]

	Age (years)	Education (years)	BDI	BAI	ANART	Y-BOCS
CTL (n = 10)	29.5 (12.1)	15.2 (1.2)	3.3 (4.3)	2.9 (1.8)	122.6 (3.8)	N/A
OCD (n = 10)	28.7 (12.1)	14 (2.2)	13.2 (4.6)	11.3 (8.6)	117.2 (8.7)	22.8 (3.1)

*CTL* control group, *OCD* Obsessive Compulsive Disorder group, *BDI* Beck Depression Inventory, *BAI* Beck Anxiety Inventory, *ANART* American version of the National Adult Reading Test, *YBOCS* Yale-Brown Obsessive Compulsive Scale

### Tasks

In the first standard Flanker Task, the overall error rate for the OCD group (16 %) was higher than that for the CTL group [10 %; t(18) = −1.4, p < 0.05]. Both OCD patients and controls performed the task with similar response times [RT for correct trials: CTL: 426.11 ms (SD 65.1), OCD:453.00 ms (SD 89.8), *t*(*18*) = −1.3, p > 0.05; RT for error trials: CTL: 344.25 ms (SD 55.7), OCD: 373.14 ms (SD 82.4), t(18) = −0.1, p > 0.05], and both groups showed a comparable flanker effect (i.e. response times were slower for incongruent than congruent trials; Table 2).

**Table 2:** Flanker task performance [mean (SD)]

	RT c (ms)	RT ic (ms)	Error total (%)	Flanker effect (%)	PES (ms)	RT error report	Error report (%)	RT error correct	Error correct (%)
Baseline (before rTMS)									
CTL (n = 10)	405.2 (70.2)	449.9 (59.5)	10.2 (4.1)	11.7 (4.7)	10.6 (10.1)	441.5 (73.1)	92.9 (10.3)	217 (50.4)	89.6 (8.4)
OCD (n = 10)	433.9 (86.7)	476.3 (93)	16.2 (12.9)	9.9 (4.6)	−3.6 (10.7)	546.6 (133.2)	59 (30.6)	258.7 (77.5)	77.1 (23.7)
After rTMS (one session)									
CTL (n = 10)	401.4 (94.4)	442.4 (76.9)	10.1 (3)	11.2 (5.5)	7.6 (11.3)	356.4 (20.5)	81.8 (40.2)	191.9 (96.9)	87.4 (16.6)
OCD (n = 10)	450.4 (43.6)	491.2 (48.9)	8.2 (6.9)	9.05 (1.9)	−2.2 (14.9)	527.8 (92.3)	63.5 (32.8)	233.52 (54.6)	76.5 (31)
After rTMS (ten sessions)									
OCD (n = 10)	424.3 (63.9)	465.4 (56.3)	6.6 (4.6)	10.2 (5.9)	−1.4 (16.1)	500.14 (96.9)	51 (44)	232.3 (82.4)	77.3 (25.3)

*CTL* control group, *OCD* Obsessive Compulsive Disorder group, *RT* response time, *PES* post-error slowing, *c* congruent trials, *ic* incongruent trials

**Fig. 1:**
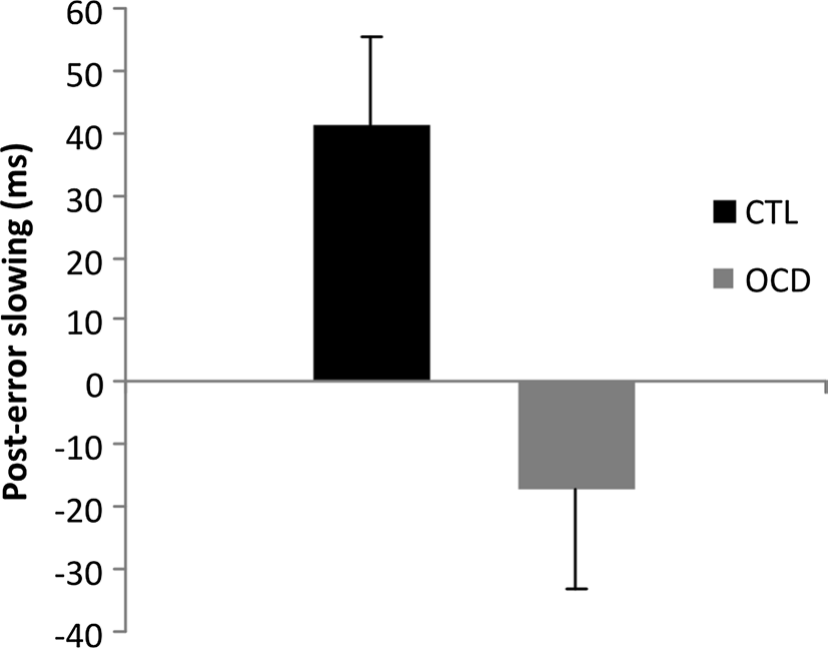
Post-error slowing. *Bars* show overall post-error slowing for the control (CTL) and patient (OCD) groups. *Error bars* represent standard deviation

Post-error slowing is calculated by comparing the mean RT of correct trials following errors with the mean RT of correct trials following correct trials. Two-factor ANOVA revealed a significant interaction of the group and the RTs for post-error and post-correct responses (F_(1,18)_ = 7.75, p < 0.05). Post-hoc analysis revealed that the CTL group demonstrated post-error slowing whereas the OCD patients did not [CTL post-error RT: 463.1 ms (SD 59.2), post-correct RT: 421.6 ms (SD 66.8), p < 0.05; OCD post-error RT: 443.3 ms (SD 78.3), post-correct RT: 460.4 ms (SD 97.6), t: 1, p > 0.05; Fig. 1; Table 2].

In a second version of the Flanker task, the two-factor ANOVA with ‘true error reports’, ‘false error reports’ and the ‘RT for reporting errors’ as “repeated measurements” and the group (OCD and CTL) as between factor showed a significant interaction between error-report variables and group (F_(2,36)_ = 7.88, p < 0.01). Subsequent post hoc analysis revealed that the OCD patients as a group reported a considerably lower proportion of their errors [OCD: 59 % (SD 31)]; CTL: 93 % (SD 10), p < 0.01; Table 2]. OCD patients were also significantly slower in reporting their errors [OCD: 546.6 ms (SD 133.2); CTL: 441.5 ms (SD 73.1), p < 0.05; Table 2]. There was no difference between groups in the proportion of false error reports [OCD: 8 % (SD 10); CTL: 4 % (SD 5), p > 0.05].

A third version of the Flanker task required participants to correct any errors, by pressing the alternate response key as quickly as possible. A two-factor ANOVA revealed no significant interaction between error-correction variable and group (F_(2,36)_ = 2.08, p > 0.05). Although the OCD group corrected fewer errors than the CTL group, the difference did not reach significant difference [OCD: 77 % (SD 24) vs. CTL: 90 % (SD 8.4), p = 0.07; Table 2]. Previous work has shown that such error correction is relatively rapid, typically occurring in 100–200 ms [35]. Both groups confirmed this pattern, correcting errors faster than their average response time [RT for error correction to average RT: OCD: 62 % (SD 13), CTL: 56 % (SD 13), p < 0.05]. Patients and CTLs showed a similar rate of false correction [OCD: 7 % (SD 8), CTL: 3 % (SD 4), p > 0.05].

Finally, to answer whether the self-report BDI and BAI scores influenced task performances in either group, we ran the above analyses one more time with BDI and BAI scores as covariates. Neither the BDI nor the BAI scores had any significant interactions with any of the task performances. We also performed linear regression analyses with BDI and BAI scores as independent variables and task performances (i.e. % error, PES, % error report, % false error reports, % error correction and % false error correction and RTs in different conditions) as dependent variables, and we found no significant correlations between the scores on these two self-report questionnaires and the above error indices (p > 0.05 in all analyses).

### The effect of one session of rTMS on error-related indices

In the next step, we looked at the effect of one session of low frequency rTMS over the mPFC on Flanker task performance by performing an ANOVA with a 2 (group) by 2 (baseline vs. post-TMS) design. The ANOVA revealed a significant interaction between group and error rate (F_(1,18)_ = 6.306, p < 0.05). A pairwise post hoc comparison showed that this interaction was driven by a drop in error rate for OCD patients from baseline to post-one session (p < 0.05; Table 2). The CTL group did not show a similar reduction in errors (p > 0.05). In the “error report” task, OCD patients continued to report fewer errors than the CTL group (repeated measure ANOVA with significant main effect of group: F_(1,18)_ = 5.734, p < 0.05). Similarly, the patients remained significantly slower than controls in reporting their errors (F_(1,18)_ = 10.49, p < 0.01; Table 2). There was no significant interaction between the groups and error correct indices before and after one session of rTMS (F = 0.238, p > 0.05).

We also compared patients’ performances (and not CTLs’) at three time points: at baseline, after one session of rTMS and after ten sessions of rTMS. We did not include the CTL data in this analysis because CTL subjects did not receive ten sessions of rTMS. Repeated measures ANOVAs did not reveal any significant differences in flanker effect, RTs, percentage of reporting errors, or percentage of correcting errors (Table 2). However, patients’ error rates showed a significant drop after one and after ten sessions of rTMS (pairwise comparison with bonferroni correction, p < 0.05; Table 2). There was a trend toward normalized PES following treatment, with RTs after correct responses (but not after error responses) dropping significantly after ten sessions (F_(2,18)_ = 7.66, paired comparison with bonferroni correction: p < 0.05).

OCD patients showed a significant reduction in Y-BOCS scores following ten sessions of rTMS, a reduction which persisted at 1 month follow-up (for details and a report of this analysis, please see [36]). For the present analysis, we wanted to see if this change in Y-BOCS scores correlated with patients’ performance on the Eriksen Flanker tasks. We found that post-correct RTs were negatively correlated with Y-BOCS scores after both one and ten sessions of rTMS (after one rTMS: r^2^ = 0.48, p < 0.05; after ten rTMS: r^2^ = 0.57, p < 0.05).

## Discussion

The present study focused on trial-by-trial error-related processes and their relationship with the clinical symptoms in OCD. It also investigated the modulatory effect of low frequency rTMS over the medial prefrontal cortex on these measures. We found that OCD patients showed an absence of normal post-error slowing, higher error rates compared to healthy controls, and impaired conscious error reporting.

The absence of PES in adult OCD in the current study is consistent with prior work in pediatric OCD showing an absence of PES for incongruent trials [17]. This suggests that impaired PES might be an early neurocognitive marker in OCD. However, studies in adults have largely reported similar error rates and normal PES between OCD patients and healthy controls [4, 12]. For example, Hajcak et al. showed that OCD patients demonstrate post-error slowing in a Stroop task with no response time limitation [4]. Furthermore, OCD patients in a study by Fitzgerld et al. showed greater slowing than controls after the error trials in a letter version of the Eriksen flanker task [12]. In Fitzgerald and colleagues’ study, the stimulus duration was 1500 ms, compared to the 100 ms stimulus duration in our paradigm. Subjects in our study were also repeatedly encouraged to respond as quickly as they could through text prompts and performance feedback. It is possible that the rapid stimulus presentation and greater pressure for a speeded response produced this difference in behaviour between studies. It may be that OCD patients are able to adapt their behaviour after an error commission in the absence of pressure for a speeded response. Conversely, in tasks that require both accuracy and speed—such as the task in the present study—they fail to demonstrate cognitive adaptation of their behaviours after errors. Differences in the response stimulus interval (RSI) might also be a reason for this discrepancy between studies. For example, it has been shown that PES is affected by the length of the RSI in healthy populations, with PES being the highest with a short RSI and absent with an RSI greater than 1500 ms [37]. In our paradigm, the RSI was very short (200 ms in the “standard” version of the task). We found that healthy controls showed PES regardless of the trial type whereas OCD patients did not. It is possible that short time allocation for OCD patients works disadvantageously, making them react differently to their errors vs. their correct responses; i.e. when a fast response is demanded, OCD patients may be unable to allocate adequate attention to their errors in order to lower their error rate.

We found a trend towards OCD patients’ reaction times being slower after correct responses than healthy controls, suggesting that the absence of PES in our OCD group is due to slowed RTs following correct responses. In line with this observation, Riesel et al. showed that in addition to ERN, correct-related negativity (CRN) is also enhanced in OCD and its amount is correlated with OCD symptoms [6]. Interestingly, Endrass et al. showed that OCD individuals with higher Y-BOCS scores showed more negative response-related amplitudes in CRN than individuals with lower scores [38]. Pronounced CRN amplitudes were also observed with high stimulus or response ambiguity [39, 40]. It is possible that increased CRN signal, similar to increased post-correct RTs in our study, is due to the uncertainty and thus compromised representations of the correct response [41]. OCD symptoms could be attributable to an existing error processing dysfunction in certain individuals. More specifically, OCD patients may suffer from inappropriate attention allocation to correct vs. incorrect actions. Washing one’s hands or locking a door, actions which would be considered correct and complete for most people, do not seem to be sufficient for an obsessive brain. Intriguingly, the proportion of false error reports in this study did not differ between the OCD group and controls. This is consistent with the clinical behavioural profile of OCD: patients maintain a conscious awareness of the fact that their actions are not literally erroneous, yet they feel compelled to repeat them anyway. Indeed, this discord between knowledge and compulsion is a major source of distress for many OCD individuals.

In clinical studies, PES has often been used as a marker for ‘cognitive control’ [42–44]. The absence of PES in OCD, which seems to be secondary to increased post-correct RTs, fits with OCD symptoms: patients will often repeat an act compulsively (e.g. washing hands or checking a lock) despite having performed the act correctly the first time. Assuming a cognitive control model for PES, it appears that OCD patients misdirect attentional resources to already correct responses. Alternatively, the ‘orienting account’ theory posits that PES is an orienting response to an unexpected event, rather than an error-driven cognitive control adjustment. Under this assumption, post-error slowing is observed when errors are infrequent, whereas slowing is observed after correct trials when errors are frequent. In the present study, the error rate amongst individuals with OCD was significantly higher than that of CTLs. Perhaps, then, the default belief for individuals with OCD is that their actions are erroneous. This leads to the perception that correct trials are infrequent and a deviation from their expectation, resulting in the observed post-correct slowing of RTs.

### The effect of rTMS on error-related processes in OCD

One of the purposes of this study was to investigate the modulatory effect of low frequency deep rTMS over the medial prefrontal cortex of patients with OCD. We found that all patients responded to 2 weeks of rTMS with a significant reduction in their symptoms, and that this effect persisted 1 month following treatment [36]. Medial prefrontal cortex is involved in both error processing and cognitive control [45]. It has been shown that damage to this region disrupts error prediction in several tasks [29]. Specifically, these patients have problems predicting the likelihood of future errors. ACC hyperactivity and hyperactive error and correct signals (demonstrated by ERN and CRN in ERPs) have been seen in OCD patients [2, 6, 12]. It is possible that the observed hyperactivity of the dACC in OCD [14] reflects a fundamental dysfunction in error processing. We found that rTMS over this area decreased post-correct slowing impairment, error rate and Y-BOCS scores. It is possible that deep rTMS over the dACC may help rectify faulty error processing, which may represent a mechanism of action against OCD symptoms. However, it is equally possible that rTMS intervention is altering a more widespread neural network or influencing other processes, which results in the independent modulation of both error processing and OCD symptomology. For example, previous imaging studies in OCD have shown greater relative activation of the supplementary motor area (SMA) during a multi-source interference task with high-conflict (incongruent > congruent) trials [46]. It has also been shown that inhibitory forms of rTMS over the SMA (which sits above the dACC), improve OCD symptoms. It is possible that the improvement in OCD symptoms in our study is related the rectifying deficient inhibitory processes mediated by the SMA. In future studies, functional imaging following rTMS treatment could further explore changes in the activity of cortical networks involved in OCD as a result of rTMS to help elucidate the mechanism of this modulatory effect.

Surprisingly, we found a strong negative correlation between post-correct RTs and Y-BOCS scores after rTMS application. In other words, patients with higher symptom severity had longer post-correct RTs. Given that both post-correct RTs and Y-BOCS scores dropped significantly following rTMS, the emergence of a negative correlation between them was surprising. This finding suggests a strong modulatory effect of rTMS on the correlation between post-correct RT and OCD symptoms. Whether these two parameters are altering each other in a compensatory fashion or whether there is a third mediator that is driving this correlation cannot yet be determined by this study. Further research exploring the nature of this correlation will be important to shed light on the relationship between trial-by-trial error parameters and OCD symptoms.

There are a few limitations in this study that we should take into consideration. The relatively small sample size and the average young age of the OCD patients who participated in this pilot study limit our ability to generalize these results to the entire OCD population. Furthermore, it is possible that the observed improvements in error rate and PES might be due to practice effects rather than a true result of rTMS application. However, the observation that OCD but not CTL subjects showed improved error rates following a single session of rTMS suggests that rTMS had an effect on patient behaviour independent of practice effects (Table 2). In addition, OCD patients did not show an improvement on other error indices following the third task-repetition; the percentage of error report, error correction, and the flanker effect did not improve with practice, suggesting that the observed selective improvement in post-correct RTs and error rate might be the direct effect of rTMS in OCD (and not CTL) subjects. Nonetheless, in the absence of a control-OCD group following the same testing procedure without rTMS intervention, we cannot unequivocally differentiate practice effects and rTMS-induced changes in behaviour.

## Conclusions

OCD appears to selectively impair the conscious reporting of errors and increase reaction times following correct responses. These observations suggest that individuals with OCD may misdirect attentional resources to already correct responses. Low frequency rTMS over the medial prefrontal cortex was found to improve abnormal post-correct reaction times and reduce overall symptoms in OCD patients, highlighting this brain region as a potential target area for treatment.

## Abbreviations

OCD: obsessive compulsive disorder
mPFC: medial pre-frontal cortex
dACC: dorsal anterior cingulate cortex
pMFC: posterior medial frontal cortex
rTMS: repetitive transcranial magnetic stimulation
ERN: error-related negativity
ERP: event-related potential
PES: post-error slowing
CNS: central nervous system
CTL: control
SSRI: selective serotonin reuptake inhibitor
SNRI: selective noradrenalin reuptake inhibitor
M.I.N.I.: Mini-International Neuropsychiatric Interview
Y-BOCS: Yale-Brown Obsessive Compulsive Scale
NART: National Adult Reading Test
RT: reaction time
EMG: electromyography
BA: Brodmann area
CRN: correct-related negativity
SMA: supplementary motor area
